# Health Monitoring of Aerospace Structures Utilizing Novel Health Indicators Extracted from Complex Strain and Acoustic Emission Data

**DOI:** 10.3390/s21175701

**Published:** 2021-08-24

**Authors:** Georgios Galanopoulos, Dimitrios Milanoski, Agnes Broer, Dimitrios Zarouchas, Theodoros Loutas

**Affiliations:** 1 Applied Mechanics Laboratory, Department of Mechanical Engineering and Aeronautics, University of Patras, 26504 Rio, Greece; gkgalan@gmail.com (G.G.); d.milanoski@g.upatras.gr (D.M.); 2 Structural Integrity and Composites Group, Faculty of Aerospace Engineering, Delft University of Technology, 2629 Delft, The Netherlands; A.A.R.Broer@tudelft.nl (A.B.); d.zarouchas@tudelft.nl (D.Z.)

**Keywords:** structural health monitoring, composite stiffened panels, FBG sensing, acoustic emission, health indicators

## Abstract

The development of health indicators (HI) of diagnostic and prognostic potential from generally uninformative raw sensor data is both a challenge and an essential feature for data-driven diagnostics and prognostics of composite structures. In this study, new damage-sensitive features, developed from strains acquired with Fiber Bragg Grating (FBG) and acoustic emission (AE) data, were investigated for their suitability as HIs. Two original fatigue test campaigns (constant and variable amplitude) were conducted on single-stringer composite panels using appropriate sensors. After an initial damage introduction in the form of either impact damage or artificial disbond, the panels were subjected to constant and variable amplitude compression–compression fatigue tests. Strain sensing using FBGs and AE was employed to monitor the damage growth, which was further verified by phased array ultrasound. Several FBGs were incorporated in special SMARTapes^TM^, which were bonded along the stiffener’s feet to measure the strain field, whereas the AE sensors were strategically placed on the panels’ skin to record the acoustic emission activity. HIs were developed from FBG and AE raw data with promising behaviors for health monitoring of composite structures during service. A correlation with actual damage was attempted by leveraging the measurements from a phased array camera at several time instances throughout the experiments. The developed HIs displayed highly monotonic behaviors while damage accumulated on the composite panel, with moderate prognosability.

## 1. Introduction

Composite materials are being increasingly used as structural components in many applications. In the aeronautics industry, modern aircraft structures consist of more than 50% composite materials [1], due to their unique property of high strength-to-weight ratio, in addition to enhanced corrosion resistance. However, the non-homogeneous nature of these materials involves complex and not yet perfectly understood degradation and failure processes. These structural components are constantly subjected to variable service loads and environmental loadings, which can greatly reduce their load-bearing capability, and may cause damage that fails to be identified during routine inspections. A good example of such damage is Barely Visible Impact Damage (BVID), which causes subsurface damage that is critical to the structure’s integrity [2]. Structural Health Monitoring (SHM) technology has been introduced and evolved during the past 30 years, with the aim to detect, locate, and quantify the degradation of a structure [3]. A recent trend has emerged that aims to achieve an even more demanding task, namely, the utilization of SHM data for the intelligent prognosis of a structure’s remaining useful life [4,5]. SHM, as utilized via a network of sensors attached strategically to a structure, can ultimately lead to the implementation of a Condition-Based Maintenance (CBM) paradigm, significantly reducing downtime-induced delays and unscheduled maintenance costs, in addition to increasing safety [6]. The SHM technologies most sought-after by the research community include Fiber Optic Sensors (FOSs) for strain sensing, piezo-electric elements for Lamb wave testing, comparative vacuum monitoring, acoustic emissions (AEs), and vibration testing.

### 1.1. State of the Art

#### 1.1.1. Fiber Optical Sensors

FOSs are a state-of-the-art sensing technology able to monitor composite structures. Their tolerance to electromagnetic interferences and environmental conditions offers significant flexibility for use in various applications. FOS, particularly Fiber Bragg Gratings (FBGs), have attracted the interest of numerous researchers and applied to various fields. Milanoski and Loutas [7] numerically studied a composite single-stringer panel with the use of virtual FBGs. They developed strain-based health indicators (HIs) for skin-stiffener disbond monitoring. Palaniappan et al. [8] used embedded chirped FBGs to monitor disbonds on adhesively bonded composite joints. It was observed that initiation of disbond growth causes a wavelength shift at the sensor located closest to that disbond. Kahandawa et al. [9] extensively studied the effects of various loading conditions on the strain readings of FBGs. They used an artificial neural network (ANN) to predict damage evolution in the structure using FBG readings as the indicator of damage. Takeda et al. [10] used embedded FBGs to monitor the central wavelength reflection spectra during impact damage on composite panels. It was shown that the wavelength spectra were permanently altered after the impact. Sbaruffati et al. [11] proposed a damage detection method using the Mahalanobis distance. The method was first implemented on a finite element model of a helicopter tail to monitor fatigue crack propagation. To validate the method, an experiment with permanently bonded FBG sensors to record strains was conducted. In addition, Sbaruffati et al. [12] proposed a methodology for crack damage diagnostics based on a normalized strain damage index. ANNs were used for the diagnostics trained based on experimental and FEM data. In another study, Guemes et al. [13] used FBG sensor readings with PCA to detect different types of damage on a composite wing spar. The T^2^ index and Q index were used to discern the different damage cases. Guemes et al. [14] used FOS (both distributed and FBG) to monitor damage in composite structures. Damage was identified, but the location and size of the damage in conjunction with the sensor location played an important role.

#### 1.1.2. Acoustic Emission

AE is also a powerful SHM solution that has been extensively used in the literature [15]. Zhou et al. [16] used acoustic emissions with digital image correlation (DIC) to monitor the compressive behavior of multi-delaminated composites. More specifically, they correlated the amplitude, duration, and relative energy with damage propagation. A novel experimental campaign was conducted by Camri et al. [17] on aluminum–GFRP hybrid specimens with mounted AE sensors. They conducted experiments at both the laboratory scale and using a scanning electron microscope in an attempt to correlate AE data with the tracking of damage evolution. The use of AEs in SHM-based prognostics of the remaining useful life has also been recently studied. Loutas et al. [4] and Eleftheroglou and Loutas [5] used acoustic emission features as damage indicators for remaining useful life prediction of composite open-hole specimens. It was observed that the windowed cumulative rise time to amplitude ratio (RA) has a monotonic trend and can be used for prediction tasks. This was also observed for the windowed cumulative energy. Liu et al. [18] used acoustic emission data to predict the remaining useful life of composite coupons under cyclic loading. It was concluded that AEs can be used monitor the degradation process, and setting an appropriate amplitude threshold significantly improved the process efficiency. In recent studies [19], specialized FBG sensors have been used for recording AEs. Despite the low sensitivity of regular FBG sensors in AE recording [20,21], these specialized phase-shifted FBGs successfully identified damage modes in three-point bending experiments, and the results were comparable to those of regular AE sensors. Broer et al. [22,23] recently proposed a methodology of fusing two SHM techniques, namely AEs and strain sensing. The idea of fusing results from several sensors was proposed in order to take advantage of the strengths of both SHM techniques, and thus obtain more effective damage diagnostics of deteriorating composite panels.

#### 1.1.3. Health Indicators

However, to exploit the direct output of a SHM system (i.e., raw sensor data), intelligent data processing and features that effectively capture the degradation process are needed. These features are usually referred to as health indicators (HIs). The quality of the HIs’ evolution over operational time largely determines the effectiveness of diagnostic systems [22] and affects the performance of the prognostics methodologies [24]. As we discussed previously in [25], HIs to be potentially used for diagnostic and prognostic tasks should possess the properties of monotonicity, prognosability, and trendability. Monotonicity characterizes a general increasing or decreasing trend of the HI, prognosability measures the spread of the HI failure values, and trendability indicates whether degradation histories of a specific structure/subcomponent have the same underlying trend. HIs can be divided into two categories, as discussed in [26,27], namely, HIs that are directly derived from physical measurements (pHIs), such as static or dynamic strains, ultrasound, temperature, or a combination of these properties; and virtual health indicators (vHIs), which are not tied to any physical properties, such as the reconstruction error (Q index) from principal component analysis. An example of a pHI can be found in [28], in which axial strain from DIC measurements was used to predict the RUL of open-hole composite coupons. Strain HIs were also developed in [29] to monitor disbond growth in composite stringer panels. VHIs are usually optimized to obtain desirable attributes, such as monotonicity, trendability, and prognosability, which significantly benefit the performance in prognostics, as postulated in [25]. Principal component analysis (PCA) is frequently used as a means to create vHIs. Loukopoulos et al. [30] applied PCA, specifically the T^2^ and Q index, as HIs to predict the RUL of reciprocating compressors. Zhang et al. [31] used wavelet packet decomposition to extract features in combination with PCA to reduce the dimensionality of the data without losing information. The output of the PCA was used as an input to a Back-Propagation ANN to predict machinery failure. In [32], an autoencoder-based Recurrent NN (RNN) is proposed for dimensionality reduction into a 2D feature space. An HI is proposed based on a distance metric and a radial basis function normalization scheme for RUL prediction of aircraft engines.

**Figure 1:**
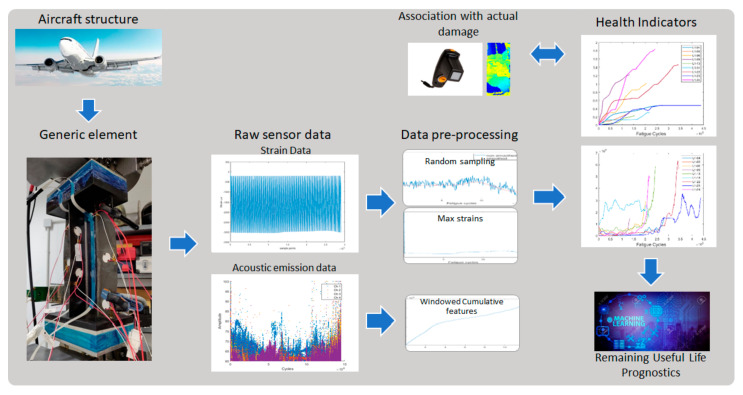
Schematic representation of the proposed methodology.

In the present work, we investigated different methods for constructing novel HIs using rather complex raw FBG strain data and AE SHM data acquired from single stringer panels (SSPs) under constant and variable amplitude fatigue testing. The objective was to identify robust and reliable HIs to use in prognostic tasks. We consider a robust and reliable HI one that has high monotonicity, i.e., evolves monotonically as damage accumulates, and high prognosability, i.e., exhibits a similar failure threshold in different test articles and is least affected by variable operating conditions. The proposed methodology is schematically shown in Figure 1. From fatigue experiments on a generic aircraft element such as the SSP, the raw sensor data were extracted from FBG and AE sensors, and then pre-processed with different approaches. The data were then further processed to develop HIs with monotonic behaviors linked to the component’s degradation. An estimation of the actual damage provided by frequent phased array C-scans was utilized to evaluate the HI’s correlation to the evolving damage. The remainder of this paper is organized as follows: Section 2 provides information on the experimental campaign and data pre-processing; in Section 3, the proposed health indicators are presented, and in Section 4 the experimental results are discussed. Finally, the main concluding remarks are provided in Section 5.

## 2. Experimental Procedure

### 2.1. Test Article Definition

**Figure 2:**
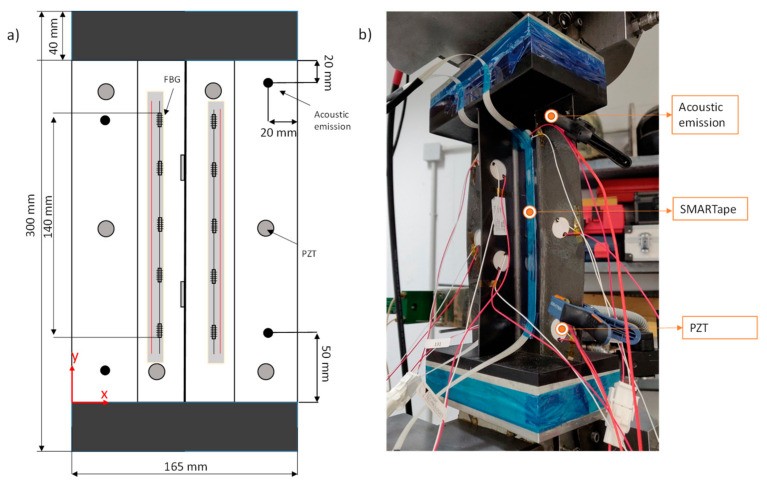
(**a**) Schematic description of the SSP and the sensor positioning, and (**b**) actual test setup.

Single stringer composite panels were manufactured by Optimal Solutions (Alcabideche, Portugal). Both the skin and the T-shaped stiffener were manufactured from IM7/8552 carbon reinforced epoxy unidirectional prepreg with [45/−45/0/45/90/−45/0]_S_ and [45/−45/0/45/−45]_S_ layups, respectively. A schematic of the panel geometry is shown in Figure 2a. Additionally, two resin tabs were placed on the specimens to ensure proper load introduction and uniform loading during the experiments. All tested specimens had some form of initial damage. In a small number of specimens, an artificial disbond was introduced during manufacturing using a Teflon insert of various sizes between the skin and the stiffener, whereas the rest were impacted at a drop tower with various energies, as analyzed in Table 1 and Table 2, and with a target to induce BVID.

### 2.2. Test Campaign

Static compression tests with a constant displacement rate of 0.5 mm/min were first conducted to determine the ultimate collapse load of the panels. The average collapse load was 100 kN and this value guided the selection of the fatigue load levels. Two test campaigns were executed in compression–compression (C-C) fatigue, with an R-ratio of 10 and a frequency of 2 Hz. The first campaign was conducted in the Aerospace Structures and Materials Laboratory of the Technical University of Delft. The specimens were tested in constant amplitude fatigue on an MTS hydraulic test machine having a capacity of 500 kN. The second test campaign was conducted in the applied mechanics laboratory of the University of Patras and the specimens were tested on variable amplitude fatigue on an INSTRON 8802 with a 250 kN load cell. The load was increased after arbitrarily selected blocks of constant loading and after checking the damage state as captured by the phased array C-scans. The details of each test campaign can be observed in Table 1 and Table 2.

Two SHM technologies were employed throughout the tests to monitor the SSPs’ structural integrity i.e., AEs and strain sensing with FBGs. AEs were recorded continuously during the experiments. In the first campaign, four Vallen VS900-M broadband sensors paired with an AMSY-6 Vallen acquisition system were used with external pre-amplification with a gain of 34 dB. In the second campaign, two Micro200HF 500–4500 kHz wideband sensors, from Physical Acoustics Corporation, with a Micro-II acquisition system, from Mistras Group, and an external pre-amplification with a gain of 40 dB, were used. The AE sensors were strategically placed on the SSP’s skin (Figure 2). A universal amplitude threshold of 65 dB was set. Because the test campaigns had different numbers of sensors, in the constant amplitude test campaign we split the AE sensors into groups of two, containing the sensors on the same vertical plane. Then, we selected the sensor group which was adjacent to the damage because this was the position of the sensors in the subsequent variable amplitude campaign. The strain measurements were not taken continuously; rather, every 500 cycles, the fatigue test was paused, and the specimens were unloaded and then quasi-statically loaded to the absolute maximum fatigue load. During the quasi-static loading, the strain measurements from the FBGs were taken using a two-channel sm130 dynamic interrogator from Micron Optics. The strain data acquisition rates were set at 100 and 5 Hz for the first and second test campaign respectively (it was observed after the first campaign that the 100 Hz acquisition rate was excessive), and the quasi-static load rate was 0.5 mm/min, which was the same as that used in the static tests. The optical fiber (OF) containing the FBGs was embedded in a SMARTape™ [33] for ease of handling purposes, which was manufactured and provided by SMARTEC (Manno, Switzerland). One SMARTape™ was adhered to each foot of the stiffener using a co-polyamide-based adhesive. The bonding quality was visually evaluated and tested for slippage. Each OF entailed five equidistant FBG sensors, for a total of ten FBGs per panel. The measurement length was approximately 140 mm focused in the middle section of the specimen, and the FBG spacing was 20 mm. The conversion from wavelength to strain was undertaken using Equation (1):

(1) $$\varepsilon =\frac{\Delta \lambda }{\lambda_{0}}*f_{g}$$

where $f_{g}=1.2$ is the grating factor provided by the manufacturer. PZT transducers were also placed on the SSPs, but were not studied in the current research. 

As previously stated, in the variable amplitude fatigue experiments, the damage was also monitored via a portable phased array camera. An initial C-scan of the damage area was performed pre-test and, at every 10,000–20,000 cycles, the test was paused and the SSP was inspected for damage growth. The outcome of this inspection, i.e., the extent of growth in the damage, informed the decision to increase the fatigue load level. The phased array images provided quantitative information on the extent of damage.

### 2.3. SHM Data Pre-Processing

**Figure 3:**
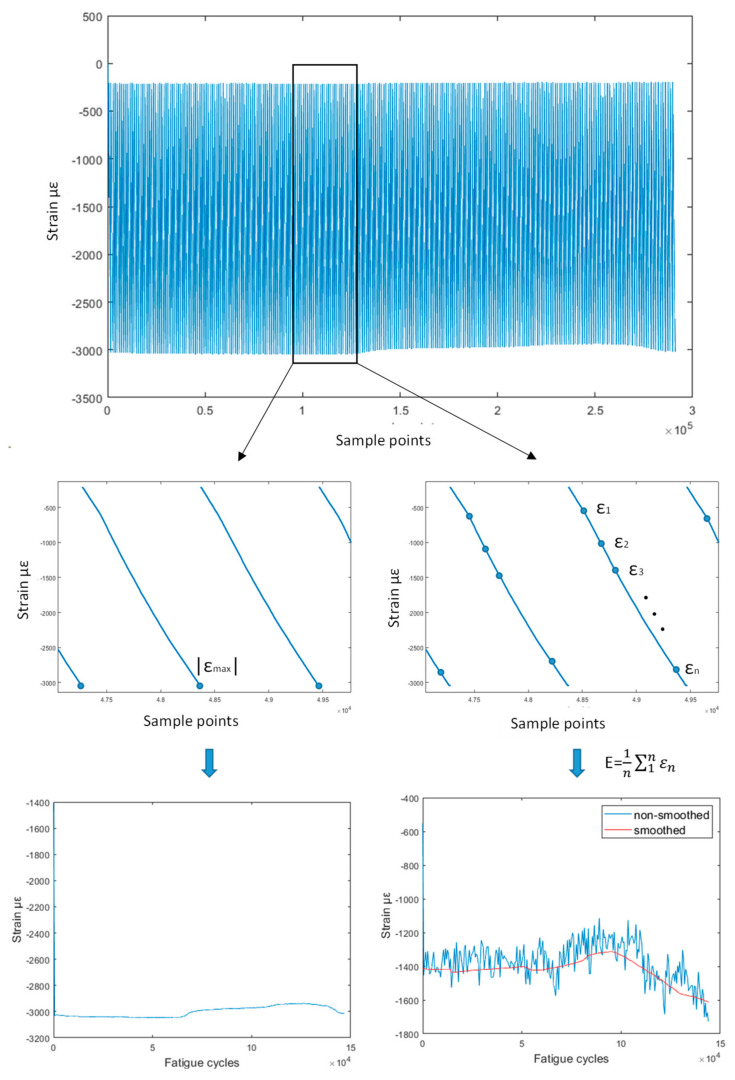
The raw strain collected at each quasistatic test is presented in the top figure. The maximum strain value from each quasistatic is extracted and creates the strain histories for method 1 (**left**). *n* strain points from each quasistatic are randomly sampled and then averaged and smoothed to create the strain histories for method 2 (**right**).

The raw SHM data and, in particular, the strain data are quite complex to visualize and are uninformative at first glance (Figure 3). Two methods were employed to pre-process the raw strain data before proceeding with the extraction of HIs. The first method utilized the peak strain values in each quasi-static experiment. This is a sensible approach in constant amplitude testing because strain evidently depends on the load. However, under a variable amplitude, which more closely resembles the real-life scenario, the peak strains change not only as the SSP is damaged, reflecting damage accumulation, but also by the operational load, a change that is not desired. Due to the load level changes in the variable amplitude campaign, steps in the peak strain at load changes were observed, which in turn negatively affect the HI evolution. The second method, which addresses this issue, involves sampling *n* random samples during the QS loading and calculating the average of these samples, as in Equation (2).

(2) $$\varepsilon =avg\left(\varepsilon_{1},\varepsilon_{2},\ldots ,\varepsilon_{n}\right)$$

Uniform distribution sampling from the entire quasi-static loading strain measurements was used. The final outcome was passed through a moving average filter for smoothing purposes. Smoothing was also applied to the variable amplitude maximum strain data to lessen the effect of the stepped behavior introduced by the load shifts, and to ensure the data was more comparable with the constant amplitude fatigue. A graphical representation of the two data processing methods is provided in Figure 3.

Regarding the pre-processing of AE data, because the quantity of data amounted to multiple gigabytes from tens of millions of AE hits, a cleaning of the data was first performed, i.e., discarding data recorded during the pauses, e.g., from the load increases during the variable amplitude fatigue experiments or other unexpected pauses. In the second stage, in addition to the classical features such as AE hits, counts, and duration, the Rise Time/Amplitude (RA) feature was calculated because it has shown interesting behavior in fatigue tests in previous work [5]. 

## 3. Methodologies—Health Indicator Development

In this section, the proposed HIs are introduced and described. Two types of HIs are developed, physical HIs, i.e., HIs obtained after simple operations on strain or AE data, and virtual HIs, i.e., HIs constructed using more sophisticated processing, such as principal component analysis (PCA). PCA is generally used to reduce the dimensionality of the data by projecting the original data into the principal component space. Each principal component is constructed by a linear fusion of the original variables using the eigenvectors and eigenvalues of the covariance matrix of the original data. PCA and its statistical quantities Q and T^2^ have been used in the literature for damage diagnostics and prognostics [13,34,35].

### 3.1. Strain Based HIs

#### 3.1.1. HI_1_

The first proposed HI was previously used in [7,36]. In these works, HI_1_ was tested on data from both QS simulations conducted in a FEM and experiments. It was observed that the HI could capture the existence of damage at increasing loads. *HI*_1_ is given by Equation (3):

(3) $$HI_{1}^{i}\left(t\right)=\frac{\left|\varepsilon_{ref}^{i}-\varepsilon^{i}\left(t\right)\right|}{\left|\varepsilon_{ref}^{i}\right|}$$

where *i* = 1, …, *N* denotes the *i*th FBG sensor number among *N* total sensors, *t* is the operational time, and $\varepsilon_{ref}^{i}$ is the strain value of sensor *i* at a reference state. Frequently, as a reference we assume the strain of a pristine specimen at the same load. However, to more closely reflect a real-life scenario, where the pristine condition or load may be unknown, we consider as reference the state at the first SHM measurement. This is the rationale behind the proposed assumption. In our case, the reference state is at the starting point for all experiments where damage was induced (impact or artificial disbond). FBG sensors positioned closer to the damage, and are thus more affected by the damage, are expected to display higher values. Moreover, HI_1_ for each sensor is only affected by measurements of the studied sensor and eliminates unwanted effects, such as missing data or abnormal behaviors, from the other sensors. 

#### 3.1.2. HI_2_

The second proposed HI was also introduced and assessed in numerical experiments in [36]. HI_2_ combines the strain measurements at all FBGs of the same foot and measures the impact of each FBG on the cumulative strain of the same foot. *HI*_2_ is defined by Equation (4):

(4) $$HI_{2}^{i}\left(t\right)=\frac{1}{n}\frac{\;\varepsilon^{i}\left(t\right)}{\sum_{1}^{n}\varepsilon^{i}\left(t\right)}-HI_{2}^{i}\left(0\right)$$

where *i* = 1, …, *n* denotes the number of sensors on the same foot and *t* is the operational time. The rationale behind HI_2_ is that sensors unaffected by damage tend to show minor deviations from the reference condition. The reference value at *t* = 0 is also subtracted from the subsequent values to allow for an equal assessment. As shown later, the strain values at all instances appeared to deviate from the reference, but sensors in the vicinity of the damage display higher deviations. HI_2_ attempts to highlight this effect. 

#### 3.1.3. HI_3_ and HI_4_

HI_3_ and HI_4_ are fused versions of HI_1_ and HI_2_ for all 10 FBG sensors, aimed at creating a single monotonic indicator. Both HI values are the root squared sum of the HI curves multiplied by an appropriate weight. As weights, the monotonicity [37] of each *HI_j_* (*j* = 1, 2) curve is used, because a major desirable attribute of every HI is a monotonic trend. The HI is squared to ensure that the output is a non-negative number. The general equation of these HIs is:

(5) $$HI_{3}\left(t\right)\;=\sqrt{\sum {\left(m_{i}HI_{1}^{i}\left(t\right)\right)}^{2}}$$

(6) $$HI_{4}\left(t\right)\;=\sqrt{\sum {\left(m_{i}HI_{2}^{i}\left(t\right)\right)}^{2}}$$

where *i* = 1, …, *N* is the number of sensors, *m_i_* is the monotonicity of each *HI_j_^i^* (*j* = 1, 2) curve, and *t* is the operational time. This fusion technique focuses on the behavior of the HI in future prognostic tasks. It forces the monotonicity attribute on the HI by assigning higher weights to the curves with higher monotonicity.

### 3.2. Virtual Health Indicators

As mentioned in the introduction of this section, the virtual HIs in the present work are created using the dimensionality reduction attribute of PCA. Other soft computing approaches, such as neural networks or unsupervised clustering, can also be used. PCA is utilized to reduce the available sensor data from 10 variables to as many as 3, depending on the explained variance retained in these components. 

#### 3.2.1. vHI_1_

The first endeavor with the virtual HIs is a modification of the indicator proposed in [32]. In the presented method, the dimensionality of the available sensor data is reduced from 10 to 2 using PCA. The Euclidian distance $d_{L}\left(t\right)=\left|\left|Z\left(t\right)-Z_{0}\right|\right|$ is calculated, where *Z*(*t*) is the vector of principal components [*PC*_1_(*t*), *PC*_2_(*t*)] for the entire lifetime and *Z*_0_ = *Z*(*t* = 0). A radial basis function is used to normalize the final HI, to ensure that it starts at 1 and fails at $vHI_{1}{}_{f}\epsilon \left[\varepsilon ,\varepsilon +\delta \right]$ with *ε* = *δ* = 0.01. The rationale of *vHI*_1_ is creating a HI that is indicative of the degradation process and, with appropriate normalization, reaches a failure point at zero. *vHI*_1_ is given in Equation (7):

(7) $$vHI_{1}\left(t\right)=\exp(-\frac{{\left(d_{L}\left(t\right)-d_{Lmin}\right)}^{2}}{\sigma_{L}})$$

where

(8) $$\sigma_{L}=-\frac{{\left(d_{Lmax}-d_{Lmin}\right)}^{2}}{2}[\frac{1}{\log_{10}\varepsilon }+\frac{1}{\log_{10}(\varepsilon +\delta )}]$$

The main drawback of *vHI*_1_ in this form is the dependence on the knowledge of $d_{Lmin}\;\mathrm{and}\;d_{Lmax}$ a priori. In real cases, these values are unknown but may be estimated from a training dataset. 

#### 3.2.2. vHI_2_

The second vHI proposed in this section is the so-called Q index or the sum of reconstructed squared residuals of PCA. This was also presented as a feature of diagnostic/prognostic potential in addition to Hotelling’s T^2^ in [30]. The methodology for calculating vHI_2_ is as follows:

- The initial portion of the sensor data matrix ***X*** considered as reference ***X****_ref_* is normalized to zero mean and unit variance. The mean ***M**_ref_* and the variance ***V**_ref_* are saved for later use.
- A PCA model is created using ***X_ref_*** and the coefficients matrix ***P**_mxn_* is calculated, where m represents the time points and n the number of sensors. The reduced matrix ***P_r_****_mxk_* is saved where k is the number of components whose explained variance is over 90%.
- Then ***M**_ref_* and ***V**_ref_* are used to scale the full dataset ***X***, and ***P_r_*** is used to transform the normalized data $\bar{X}$ to the PC space as in ***T*** *=* $\bar{X}P_{r}$.
- Then, the reconstructed matrix ***X_r_*** is calculated where ***X_r_** =* $P_{r}^{T}$***T*** *+ **R*** and ***R*** is the reconstruction error.
- *Q* is calculated in Equation (9):
  (9) $$Q\left(t\right)=\overset{N}{\underset{1}{\sum }}{\left(x_{i}\left(t\right)-x_{r_{i}}\left(t\right)\right)}^{2}$$
  where *i =* 1, …, *N* is the *i*th FBG sensor, and $x_{i}\left(t\right)$ and $x_{r_{i}}\left(t\right)$ the original and reconstructed data, respectively, at time *t.*

Hotelling’s T^2^ was also investigated but it did not provide any useful information and was not further explored. The Q index, as shown in Section 4, displayed a monotonically increasing behavior over the test time, starting from values close to zero. 

### 3.3. Acoustic Emission Based HIs

A simpler approach was adopted for the HI extraction from the huge amount of raw AE data after each fatigue test. Typically, the AE hits resulting from fatigue tests of SSPs were in the order of 1–15 million. As suggested in [28], windowed cumulative features were extracted from hits and RA. As windowed cumulative features, Loutas and Eleftheroglou in [5] defined the sum value of the feature at a specified time window. In this manner, the randomly recorded AE features obtain a periodic character of values acquired at each time window, which is more appropriate for SHM purposes. A variety of different window lengths was tested, and after several trials it was concluded that 500 cycles is a good selection for the window size for the aforementioned test campaigns for two reasons. First, this provides a satisfactory increasing trend, which is desired for both diagnostic and prognostic purposes. Second, when using 500 cycles as a window, it is possible to directly compare and possibly fuse the AE data with the strain data from FBGs, because they now share the same measurement interval. *HI_AE_* at any operational time *t* is thus calculated as in Equation (10):

(10) $$HI_{AE}\left(t\right)=\overset{t}{\underset{i=t-T}{\sum }}F\left(i\right)$$

Equation (10) starts calculating the *HI* for *t* ≥ *T*. *T* denotes the fixed time window and *F* is any *AE* feature.

## 4. Results and Discussion

In the following, the application of the methodologies presented in Section 3 is deployed in the raw SHM data acquired during the two executed test campaigns. To better demonstrate the potential of each HI, we separate the results presentation from each campaign and show the proposed HIs for one representative SSP in Section 4.1 and Section 4.2 for constant and variable amplitude fatigue, respectively. Then, in Section 4.3, results for all tested coupons are shown in common figures and for the most prominent HIs only.

### 4.1. Constant Amplitude Fatigue

#### 4.1.1. Strain-Based HIs

**Figure 4:**
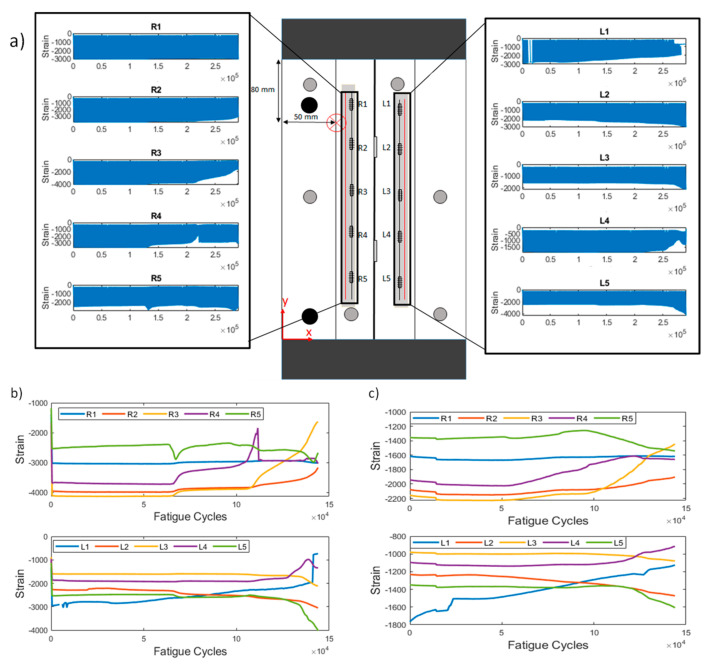
Constant amplitude test specimen CA-2. (**a**) Sensor and damage locations and raw strain data for each FBG. R and L stand for right and left, respectively; (**b**) maximum strain extracted from each quasi-static loading vs. cycles (pre-processing method 1); (**c**) random strains extracted from each quasi-static loading vs. cycles (pre-processing method 2).

As mentioned in Section 2.3, two means of raw data processing were implemented. The first retains the maximum absolute values at each QS loading (method 1), whereas the second is random sampling throughout the QS and calculating the average value (method 2). The second method is implemented in order to simulate randomness in the data acquisition, e.g., in stochastic rather than deterministic loading, and eliminate the effects of variable loading conditions (second test campaign). In Figure 4a, the locations of initial damage and the sensor positioning can be observed. The recorded strain at each sensor throughout a representative test are also presented. 

Figure 4c shows the extracted strains in a representative test of the first test campaign (CA-2) using pre-processing methods 1 and 2. A first observation concerns the different range of strain values. This is anticipated, because the strains extracted from the second method are the average of a uniform sampling of the whole QS, whereas the first method only uses the absolute maximum. Second, it can be noted that the general trend of the strains remains similar in both cases. It is also worth noting that most sensors remain rather unaffected by damage, i.e., the strain remains rather constant throughout the test time. The strain field around a discontinuity is only locally affected. Sensors R3 and R4 show an increase in their values, which indicates that damage propagating to their vicinity is affecting their measurements. 

**Figure 5:**
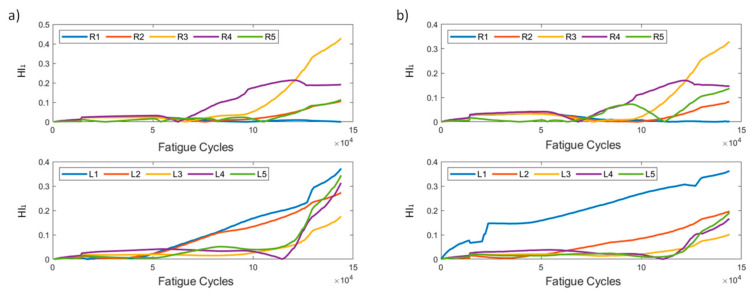
HI_1_ progression through test cycles for the two pre-processing methods for specimen CA-2: (**a**) method 1, (**b**) method 2.

In Figure 5, HI_1_ for the two methods is presented. It can be seen that, in most instances, the affected sensors display a generally increasing behavior, with the exception of R1, which shows constantly low values, suggesting that the strain field near R1 remains unaffected compared to the reference state (*t* = 0). The increasing values of HI_1_ on all sensors is also partially attributed to the coupon’s overall stiffness degradation. It is evident, however, that both methods produce similar results, demonstrating the HI’s robustness regardless of the pre-processing method. It is also worth noting the behavior of R5 in pre-processing method 2. We observe a sudden drop at 100k cycles, which is also seen in the raw strain data. This is attributed to the random sampling and smoothing during the pre-processing, and does not represent any actual damage initiation or propagation.

**Figure 6:**
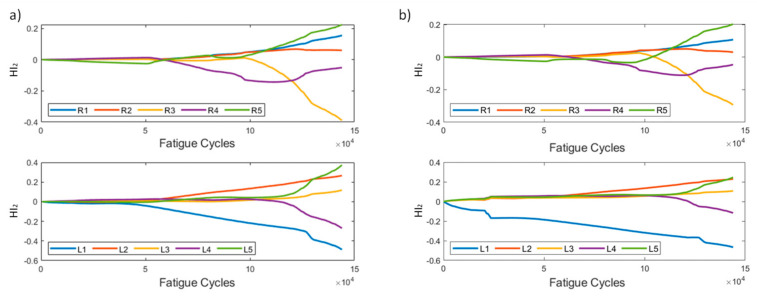
HI_2_ progression through time for the two pre-processing methods for specimen CA-2: (**a**) method 1, (**b**) method 2.

HI_2_ is shown in Figure 6. It is worth noting its behavior in the early stages. It can be seen that, until almost 50k cycles, the values of most instances are low, suggesting that, until that time, none of the sensors is greatly affected by damage; hence, there is little effect on the respective foot’s cumulative strain. After the 50k cycle mark, we observe that sensors affect the cumulative strain in different ways. It is worth pointing out that sensors R3 and L1 exhibit the largest effect on the cumulative strains on their respective feet. R2, the sensor closest to the initial damage, displays the least amount of deviation, meaning that the strain field around the sensors remained almost unaffected. Moreover, we can comment on the consistency in the HI’s behavior using the two pre-processing methods.

**Figure 7:**
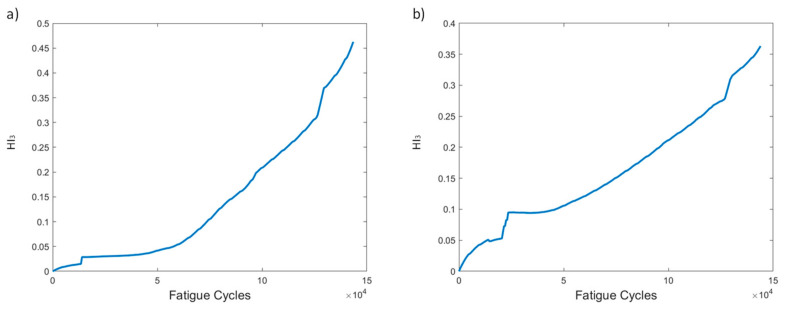
HI_3_ progression through time for the two pre-processing methods for specimen CA-2: (**a**) method 1, (**b**) method 2.

HI_3_, as stated earlier, is an attempt to create a single HI curve by fusing the ten curves of HI_1_ obtained for each sensor. The behavior of HI_3_ can be seen in Figure 7. A monotonically increasing trend can be observed in both versions of HI_3_, highlighting its main advantage. When using processing method 1, the increasing behavior is slightly faster and more consistent than when using method 2. However, both methods provide excellent increasing trends, demonstrating the good performance of HI_3_ despite the pre-processing method employed.

**Figure 8:**
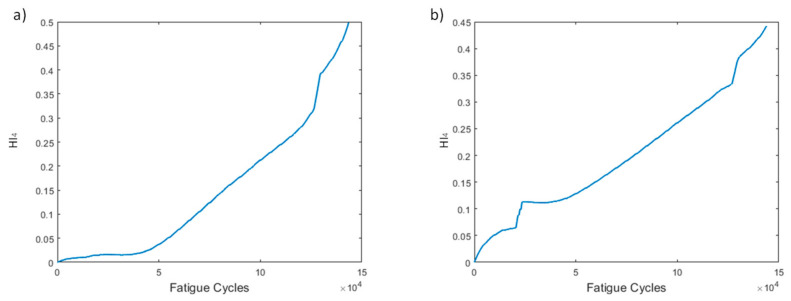
HI_4_ progression through time for the two pre-processing methods for specimen CA-2: (**a**) method 1, (**b**) method 2.

HI_4_ (Figure 8) displays a similar trend to HI_3_. Once again, both methods provide curves with increasing behaviors over time. Both trends are highly monotonic; however, pre-processing method 2 shows sudden jumps, which are attributed to the behavior of sensor L1. 

**Figure 9:**
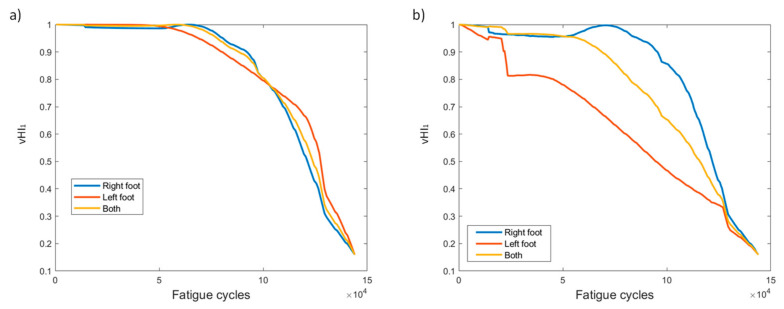
vHI_1_ progression through time for the two pre-processing methods for specimen CA-2: (**a**) method 1, (**b**) method 2.

The first of the virtual health indicators, vHI_1_, is presented in Figure 9. At first, the HI was calculated separately for each foot to observe the differences in behavior. The observation, as shown more clearly in Figure 9b, is that the HI is affected by “aggressive” behaviors, like that from sensor L1. As evidenced in Figure 4c, sensor L1 shows an “aggressive” increase in values unlike that of any other sensor. This is also projected in vHI_1_, where the left foot instance of method 2 decreases faster than in method 1. Hence, we investigated the effect if the indicator is calculated jointly for both feet, i.e., instead of calculating vHI_1_ for left and right foot FBGs separately, it was calculated for the combination of the ten FBG sensors. The result displays a more gradual behavior than that of the left foot, while also retaining aspects of both feet. This was also the case for the remainder of the specimens. To focus on the results of Figure 9, we observe that the indicator decreases gradually from 1 to 0.2 when the specimen fails. The consistency between the two pre-processing methods is slightly diminished; however, due to the nature of vHI_1_, a desirable monotonic behavior is achieved with both methods.

**Figure 10:**
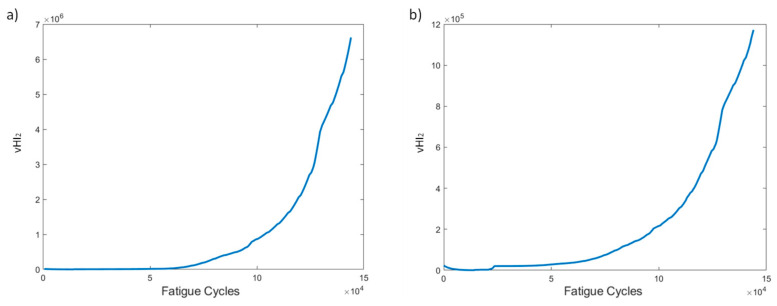
vHI_2_ progression through time for the two pre-processing methods for specimen CA-2: (**a**) method 1, (**b**) method 2.

VHI_2_ is the PCA sum of the reconstructed residual squared error, i.e., the Q index, and is depicted in Figure 10. At a first glance, we note the difference in the range of vHI_2_ values between the two pre-processing methods. Method 1 has much larger values than method 2. This is to be expected, and was previously observed, due to the higher original strain values used for the PCA model. Other than the scale differences, the two curves display similar trends of very high monotonicity, gradually increasing in time, which is a promising result for use in failure prognostic tasks. 

#### 4.1.2. AE-Based HIs

**Figure 11:**
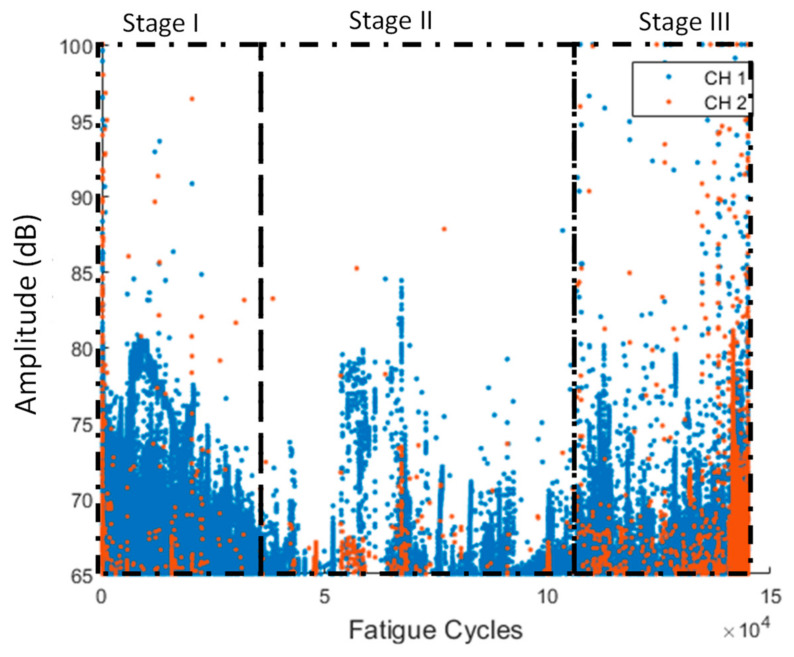
Amplitude vs. fatigue cycles for all the different AE channels for specimen CA-2 (stages are qualitatively drawn).

As we mentioned previously, AEs were constantly recorded throughout the total duration of the tests. In Figure 11, the amplitude vs. cycles can be observed. Initially, AE is high because the initial damage quickly starts to propagate (Stage I). After the first evolution, the initial damage is better accommodated by the structure through stress redistribution and does not grow significantly, reducing the acoustic activity until later stages of the experiment (Stage II). In the final stage (Stage III), the damage is starting to grow once more, leading to the final failure, which is indicated by the extensive acoustic activity.

**Figure 12:**
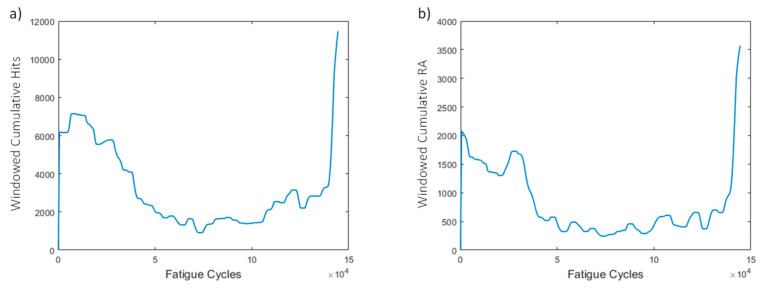
(**a**) Windowed cumulative hits and (**b**) RA for window size of 500 cycles for specimen CA-2.

Two HIs are extracted from the acoustic emission data: the windowed cumulative hits and RA. The resulting curves (Figure 12) display an increasing trend, especially near the end of life. In the early stage, both features display slightly higher values due to the early damage growth, and in the middle stage a drop in values is observed, and thereafter the values remain almost constant. Near the end of life (EoL), the HIs display a sudden increase, which is also visible in the amplitude graph. It is evident that this specimen does not have high monotonicity, but it is shown later that other specimens display more promising behaviors. However, worth mentioning again is that, near the EoL, an increasing trend is observed.

### 4.2. Variable Amplitude Fatigue

#### 4.2.1. Strain-Based HIs

**Figure 13:**
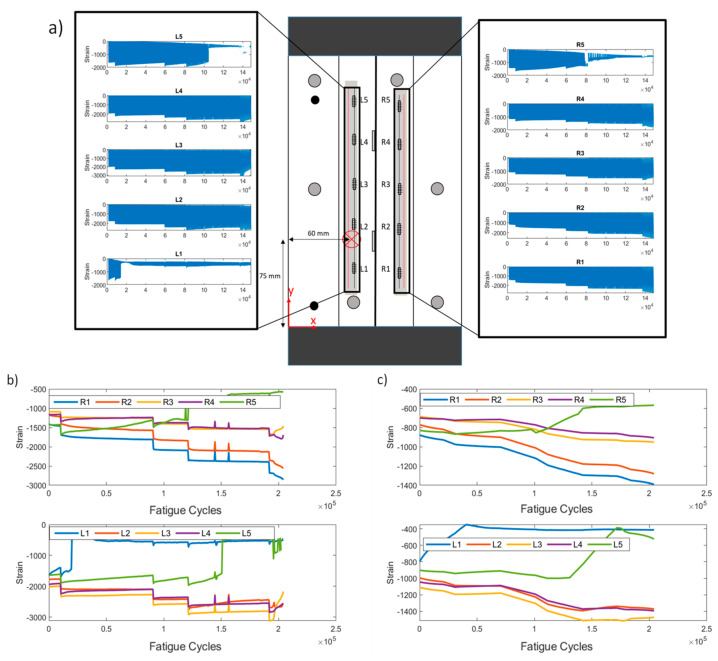
Variable amplitude test specimen VA-1. (**a**) Sensor and damage locations and raw strain data for each FBG; (**b**) extracted maximum data from each quasi-static vs. cycles (pre-processing method 1); (**c**) extracted mean data for each quasi-static vs. cycles (pre-processing method 2).

Similar to the constant amplitude test campaign, the same data processing techniques were applied and are presented. A representative specimen (VA-1) is shown in Figure 13a with the sensor positioning and the initial damage (impact) location. The data recorded at each sensor are also presented. The load increases are evident by the increase in the strain values. Moreover, it is important to note that sensor L1 is disbonded from the early stages of the test, whereas L5 and R5 disbond near the final stage. This can be deduced from, in addition to visual inspection, the significant change in the strains. These data were considered in the following steps to display the robustness of our HIs. The strains extracted using the two processing methods can be seen in Figure 13b,c. 

In method 1, the load changes can be clearly seen at the discontinuities (jumps) in strain values. This behavior would definitely affect the HI performance, resulting in instantaneous changes that may be erroneously attributed to damage. By comparison, method 2 alleviates this behavior, thus proving that the main objective for introducing this method is achieved. To provide a clearer comparison with the constant amplitude test analyses, the curves from method 1 are also smoothed before extracting the HIs. This eliminates the load effect but, as has been observed in the constant amplitude fatigue, the HIs appear to be robust, showing similar results regardless of the input data.

**Figure 14:**
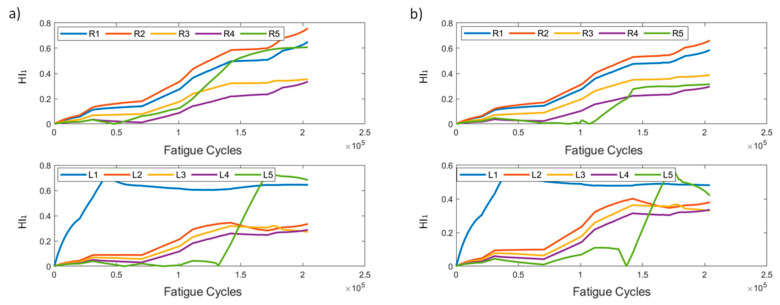
HI_1_ progression through time for the two pre-processing methods for specimen VA-1: (**a**) method 1, (**b**) method 2.

HI_1_ for both methods is presented in Figure 14. As was previously stated, the two methods have slight differences in scale, with method 1 exhibiting larger values. The general behavior of the HI remains the same in both data processing methods, and HI_1_ displays an increasing behavior over time. 

**Figure 15:**
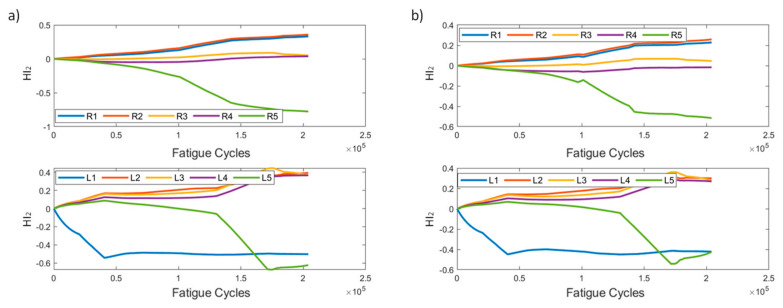
HI_2_ progression through time for the two pre-processing methods for specimen VA-1: (**a**) method 1, (**b**) method 2.

HI_2_ (Figure 15) displays similar trends as those seen in the constant amplitude fatigue campaign. The HI is slowly increasing until the 40k cycle mark, where the curves for sensors R1, R2, and R5 start to rapidly diverge, whereas R3 and R4 remain constant throughout the entire lifetime. The left foot sensors increase until 40k cycles, and then remain constant until 130k, where L1–L3 increase and L5 starts to decrease. In method 2, the right foot sensors display significantly lower values than their method 1 counterparts, but the overall behaviors are similar. Even prior to disbanding, R5 and L5 contribute most to the cumulative strains. 

**Figure 16:**
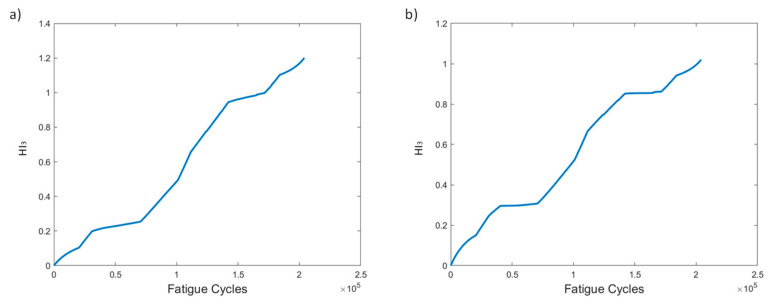
HI_3_ progression through time for the two pre-processing methods for specimen VA-1: (**a**) method 1, (**b**) method 2.

HI_3_ (Figure 16) shows almost identical behavior in both methods. A monotonous trend increasing over time, with a small number of constant slopes at the beginning and near the end, is shown. HI_3_ shows larger values when using method 1 for preprocessing, an expected outcome because the max values of the strain are higher. Once again, the main attribute of the HI, i.e., monotonicity, is achieved.

**Figure 17:**
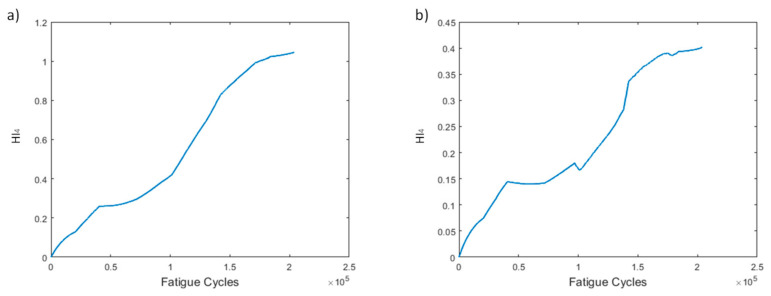
HI_4_ progression through time for the two pre-processing methods for specimen VA-1: (**a**) method 1, (**b**) method 2.

Similar trends can be observed between HI_3_ and HI_4_ (Figure 17). Increasing overall values during the specimen’s lifetime with method 1 display a slightly smoother increase. Method 2 leads to lower HI values for the first time, a fact attributed to the effect of sensor R5, which for HI_2_ shows the highest monotonicity, and hence has the largest effect in the fusion, yet shows significantly lower values than its method 1 counterpart. However, this does not affect the overall performance of H_4_, which is quite promising. 

**Figure 18:**
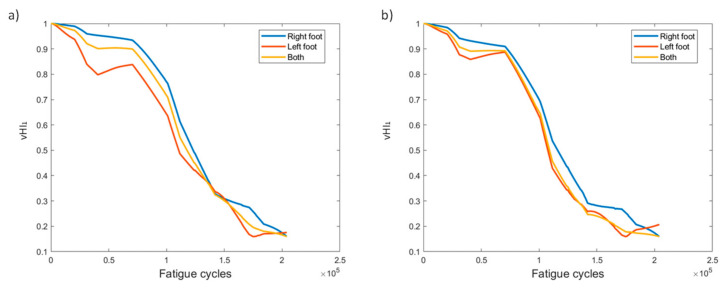
vHI_1_ progression through time for the two pre-processing methods for specimen VA-1: (**a**) method 1, (**b**) method 2.

VHI_1_ displays a gradually decreasing behavior from 1 to 0.2 (Figure 18). Again, using all sensors to calculate the HI provides a similar behavior to the average of the two feet independently and, in this particular specimen, eliminates the increase observed at the final moments in the left foot case. A good trend overall is displayed when using this HI in variable amplitude fatigue, regardless of the data processing method.

**Figure 19:**
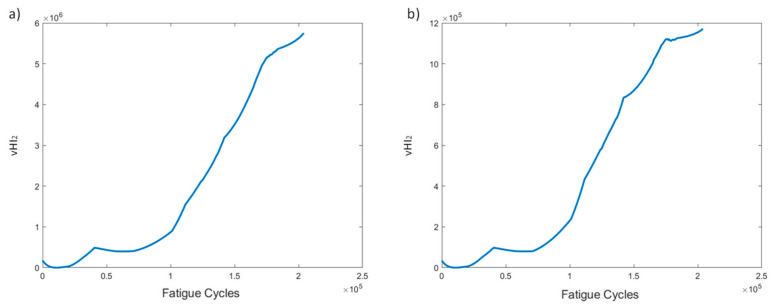
vHI_2_ progression through time for the two pre-processing methods for specimen VA-1: (**a**) method 1, (**b**) method 2.

VHI_2_ shows an increasing trend over time (Figure 19). The major difference between the two pre-processing methods is the scale of the HI. Other than this, no major differences are noted. A gradually increasing trend, which increases faster after 60k cycles, is observed. It should be noted that this indicator provides a promising trend for prognostics, regardless of the processing method and experimental conditions.

**Figure 20:**
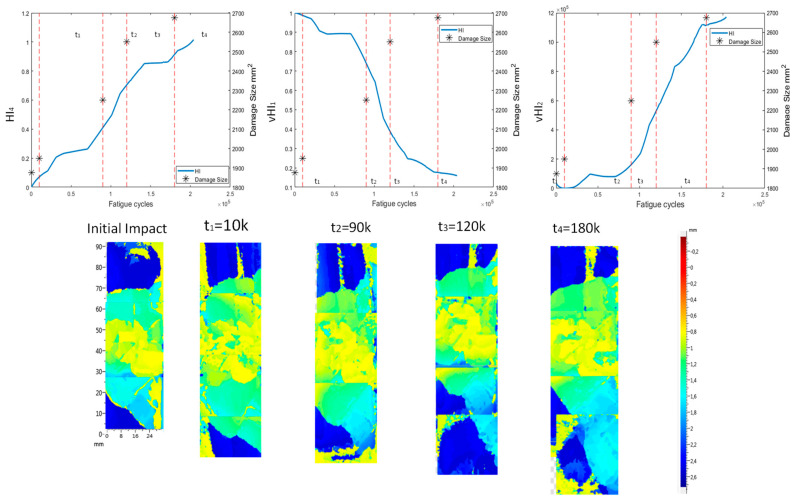
Phased array C-scan images at various instances of the VA-1 experiment, in addition to HI_3_, vHI_1_, and vHI_2_ progression through time. The dashed lines indicate the time of measurement.

The first step to evaluate the suitability of the various HIs is to determine how well they correlate with damage growth. For this purpose, we utilize the evidence acquired by the phased array C-scans that were performed at several occasions throughout each test. The three single curve HIs accompanied by the phased array images are used. The images depict the damage area, i.e., the location of the impact on the stringer’s foot. Moreover, to further demonstrate the capabilities of the HIs (HI_4_ is not presented because it displays similar behavior to HI_3_), in addition to the data pre-processing methods, method 2, i.e., the random sampling method, is used for this visualization. The damage size was also approximated using ImageJ [38]. ImageJ can measure pixel density and correlate pixel count with a known distance. From Figure 20, it is evident that there is correlation between the damage growth and HIs. The first phased array image represents the time immediately after the impact was performed, i.e., at 0 cycles. After 10k cycles, it can be observed that the damage has only slightly grown and was captured both by HI_3_ and vHI_1_. Indicator vHI_2_ at 10k cycles still displays values close to 0. The large increase in the HIs’ values after the 10k mark is attributed to the increase to the load. At 90k cycles, it is evident that the damage has grown (tail at the bottom side is larger) and this is also depicted in the HIs’ behaviors. At this point (90k cycles), the HIs start to rapidly increase in contrast to previous time points, where the increase, and the damage growth, was much slower. From 90 to 120k, the tail of the impact damage continues to grow and a rapid change in the HIs’ values accompanies this growth. In the final image, the damage growth is significant, yet it is still contained in the bottom area. The damage has grown both horizontally and vertically, which was not the case previously (the growth was mostly vertical). The HIs’ increase has declined in rate, meaning that, when reaching that point, the damage has already grown and the growth is slower during the final cycles before catastrophic failure (collapse).

#### 4.2.2. AE Based HIs

**Figure 21:**
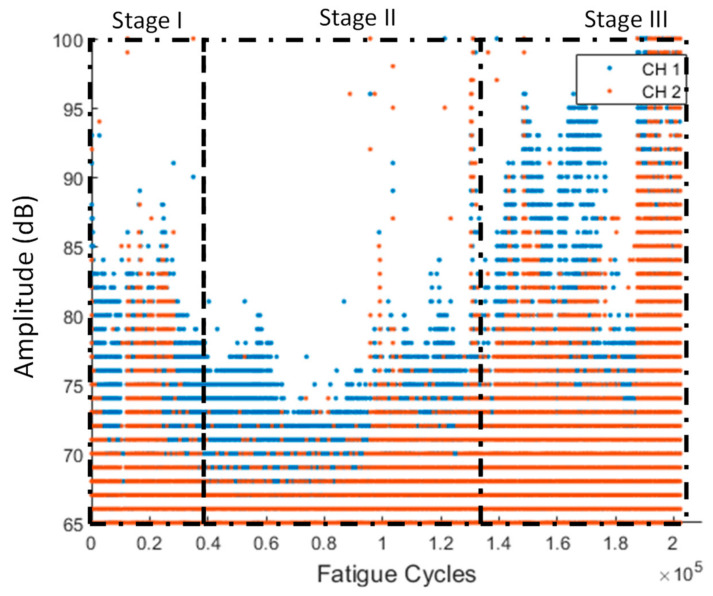
Amplitude vs. fatigue cycles for specimen VA-1 (stages I–III are qualitatively drawn).

**Figure 22:**
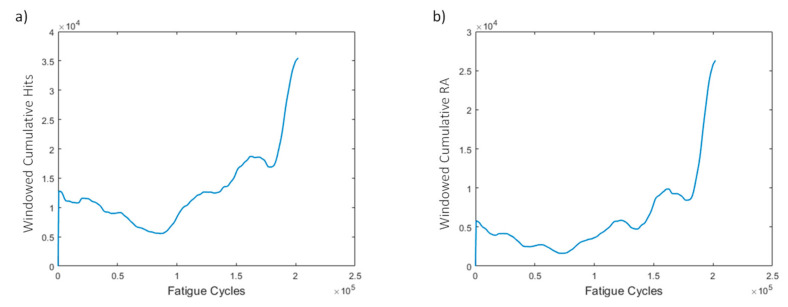
(**a**) Windowed cumulative hits and (**b**) RA vs. fatigue cycles for specimen VA-1.

The AE amplitude versus the fatigue cycles for a representative test can be seen in Figure 21. Acoustic activity presents a constant band between 65 and 72 dB throughout the entirety of the experiment, suggesting that the pre-existing damage produces significant acoustic emissions at these levels. Moreover, it is worth noting the slightly higher amplitudes in the early stages. Similar to the case of the constant amplitude fatigue, even at lower loads, the pre-existing damage appears to cause some shifts between the broken layers in the composite, although not enough for the damage to grow (Stage I). Near the EoL (Stage III), the AE amplitude is significantly increased, particularly during the final moments of the experiment, where a constant amplitude band from 65 to 100 dB is seen. Very high amplitude hits are more abundant in Stage III. Because AE hits are randomly generated and recorded, a means of obtaining periodically distributed features is to apply windowing and cumulate the AE feature(s) of interest in each window. The windowed cumulative features (hits and RA = Risetime/Amplitude) employed as HIs display an increasing trend over the course of the specimen’s lifetime (Figure 22). A slowly decreasing behavior can be seen in the early stages where initial damage propagates rapidly in Stage I, followed by a stabilization and a rapid increase towards the later stages of the fatigue experiments. Both windowed cumulative hits and RA display promising attributes and constitute potential candidates for diagnostic and prognostic purposes.

### 4.3. Discussion

**Figure 23:**
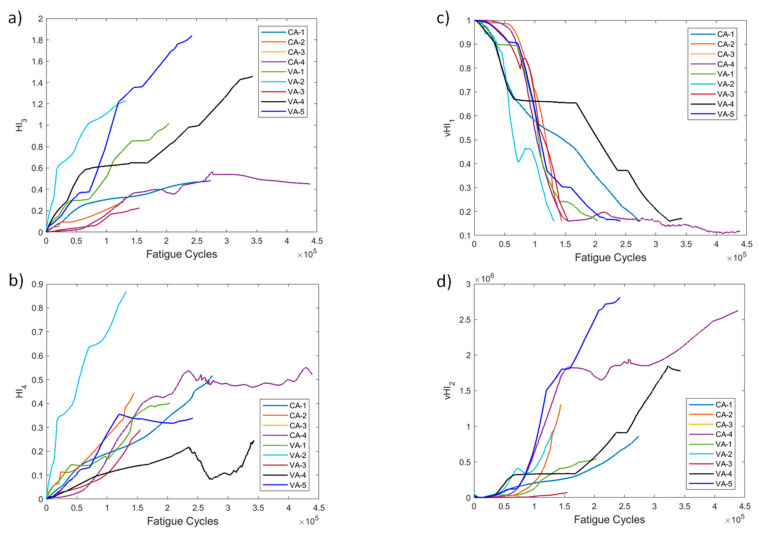
(**a**) HI_3_, (**b**) HI_4_, (**c**) vHI_1_, (**d**) vHI_2_ comparisons for all tested specimens.

Several HIs for both constant and variable amplitude fatigue were developed and their performance was presented throughout fatigue tests. The strain-based HIs, i.e., HI_1_ and HI_2_, were used in [7] to successfully detect damage in quasi-static numerical tests. The HIs investigated in the present work are intended for diagnostic and prognostics tasks in which monotonic trends are highly desirable; hence, the proposal of the fused HIs. In Figure 23a–d we nominate and present the four most interesting HIs, i.e., HI_3_, HI4, vHI_1_, and vHI_2_. These are presented comparatively for the specimens of both experimental campaigns. Because, as we previously noted, both data processing methods provide similar results, method 2 is used hereafter. The random sampling method is preferred for addressing the effects of different loads and displaying the robustness of these HIs in different loading conditions. HI_3_ and HI_4_ are shown in Figure 23a,b. Monotonicity is achieved in all specimens throughout the lifetime. Although monotonicity and similar behaviors (trendability) are highly desirable attributes, a concern is presented in the form of a lack of common failure values (i.e., moderate prognosability). Similar attributes are observed in the behavior of vHI_2_, i.e., high monotonicity but moderate prognosability. However, higher variability is observed in the vHI_2_ index due to the much higher overall values. vHI_1_ displays a decreasing trend whose range is restricted by the radial basis function normalization. It should be noted however, that vHI_1_ requires a priori knowledge of the minimum and maximum values for the normalization. It is presented as a concept due to the relatively promising decreasing trend it possesses. As previously stated, a solution to this drawback is the inference of minimum and maximum values from a training dataset.

Recently, interest has been shown in thickness strain measurements with embedded FBGs [39]. Embedding sensors into composite materials is a highly interesting concept, and may significantly improve the sensitivity of the sensors, in addition to the HIs, and also provide extra information regarding the degradation from the out-of-plain strains. Moreover, there is a possibility of developing new out-of-plain HIs to further enhance the monitoring capabilities. However, there are two major concerns regarding the sensors themselves. Firstly, the embedded sensors may compromise, even at the slightest, the structure’s integrity, and would thus be hard for the aerospace industry to adopt. Secondly, the question arises about what would happen if an embedded sensor failed. A failed sensor would be extremely difficult to replace, particularly if it was part of a larger sensor network, and replacing it would require replacing an entire part of the structure at the cost of time and money.

The strain-based HIs, combined with the data pre-processing techniques, provided overall good monotonicity for all specimens in the current study, highlighting their ability to do so despite the different loading scenarios in the two test campaigns. This also shows the robustness of the proposed HIs. Regarding HI_2_, in the pre-fused form it lacks trendability because every sensor displays a different behavior. Opposing trends are evidenced, depending on the sensor studied. Even looking at the same sensor on two different specimens, it is not guaranteed that the behavior will be similar because the buckling modes may also affect the local behavior. However, this HI can provide useful information regarding the damage progression, as seen in [7], by successfully monitoring disbond growth. It is worth pointing out that HI_2_ curves start to diverge when damage becomes significant. This can prove useful as an alarm of when to launch the prognostic task.

**Figure 24:**
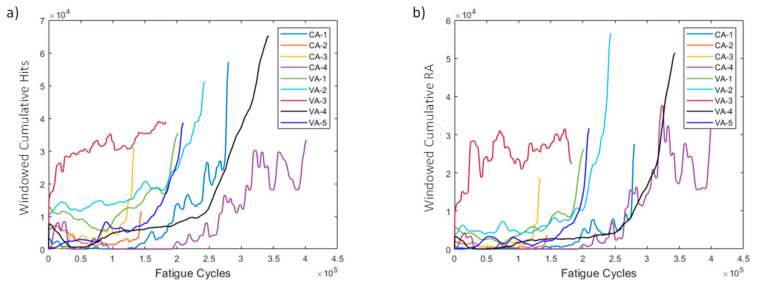
(**a**) Windowed cumulative hits and (**b**) windowed cumulative RA for all specimens.

AE-based HIs are presented in Figure 24 for all specimens of the study. Most specimens display increasing behaviors over time for both cumulative hits and RA. Interestingly, and unlike the strain-based HIs, the failure values of the AE-based HIs have less scatter (higher prognosability), making it easier to set a failure threshold compared to strain-based HIs. Not all specimens, however, are able to reach this threshold. Nonetheless, in prognostics a variety of degradation histories is necessary for greater accuracy and to account for more degradation scenarios. 

## 5. Conclusions

In the present work, several new health indicators were proposed and extracted from raw acoustic emission and strain data that was recorded after two fatigue test campaigns of stiffened composite panels. Constant and variable amplitude compression–compression fatigue tests were conducted with quasi-static loadings performed periodically. Two strain-data pre-processing strategies were examined to evaluate and demonstrate the performance of the various health indicators under different loading conditions. The random sampling across the quasi-static loading proved equally efficient in producing highly monotonic HIs while eliminating variable loading effects (discontinuities in the HI behavior).

Four physical and two virtual HIs based on PCA were proposed to extract indicators from the raw strain data. The proposed HIs displayed monotonic trends, mostly increasing throughout the specimens’ lifetime, and correlated with the degradation process as verified by the phased-array C-scans. It was also shown that the HIs have increased trendability, i.e., they show similar behaviors for both groups of tested specimens, despite the different loading conditions. Moreover, HI_3_, HI_4_, vHI_1_, and vHI_2_ displayed highly monotonic behaviors, which are highly desirable for prognostics, and HI_2_ can provide an alarm threshold for prognostics initiation. The main drawback of the strain-based HIs is the relatively poor prognosability, which makes it challenging to set a universal failure threshold. 

Two acoustic emission-based HIs were extracted, i.e., windowed cumulative hits and windowed cumulative RA. Both HIs possess lower monotonicity than the strain-based HIs but displayed higher prognosability. AE-based HIs are independent of the loading conditions. From the discussion above, and the advantages and disadvantages of both groups of HIs, it is apparent that the proposal of fused HIs (feature-level data fusion) has the potential to enhance the advantages and alleviate the disadvantages. Undertaking this research is in our plans for the near future, in addition to the implementation of the proposed HIs for evaluation of the remaining useful life.

## Figures and Tables

**Table 1:** Constant amplitude fatigue test campaign details.

Specimen #	Impact Location/Disbond	Impact Energy/Disbond Size	Load		# of Cycles to Failure
			Min.	Max.	
CA-1	Skin	10 J	−6.5 kN	−65 kN	280,098
CA-2	Stiffener foot	10 J	−6.5 kN	−65 kN	144,969
CA-3	Stiffener foot	10 J	−6.5 kN	−65 kN	133,281
CA-4	Stiffener foot	30 mm	−5.0 kN −6.0 kN ^1^	−50 kN −60 kN ^1^	438,000

^1^ load increased after 100k cycles.

**Table 2:** Variable amplitude fatigue test campaign details.

Specimen #	Impact Location/Disbond	Impact Energy/Disbond Size	Load		# of Cycles
			Min.	Max.	
VA-1	Stiffener foot	7.4 J	−4.0 kN −4.5 kN −5.0 kN −5.5 kN −6.0 kN	−40 kN −45 kN −50 kN −55 kN −60 kN	10,000 80,000 30,000 70,000 12,300 **202,300**
VA-2	Stiffener foot	10 J	−4.0 kN −4.5 kN −5.0 kN −5.5 kN	−40 kN −45 kN −50 kN −55 kN	10,000 80,000 90,000 63,000 **243,000**
VA-3	Stiffener foot	10 J	−4.0 kN −4.5 kN −5.0 kN	−40 kN −45 kN −50 kN	10,000 177,000 30,000 **217,000**
VA-4	Stiffener foot	30 mm	−3.5 kN −3.9 kN −4.5 kN −5.0 kN −5.5 kN −6.0 kN	−35 kN −39 kN −45 kN −50 kN −55 kN −60 kN	10,000 10,000 10,000 170,000 85,000 60,000 **345,000**
VA-5	Stiffener foot	7.37 J	−4.0 kN −4.5 kN −5.0 kN −5.5 kN −6.0 kN	−40 kN −45 kN −50 kN −55 kN −60 kN	20,000 75,000 25,000 62,000 60,000 **242,000**

## Data Availability

Data sharing is not applicable.
